# Protein dynamics and conformational selection in bidirectional signal transduction

**DOI:** 10.1186/1741-7007-10-2

**Published:** 2012-01-25

**Authors:** Ruth Nussinov, Buyong Ma

**Affiliations:** 1 Basic Research Program, SAIC-Frederick, Inc., Center for Cancer Research Nanobiology Program, NCI-Frederick, Frederick, MD 21702, USA; 2 Sackler Institute of Molecular Medicine, Department of Human Genetics and Molecular Medicine, Sackler School of Medicine, Tel Aviv University, Tel Aviv 69978, Israel

## Abstract

Protein conformational dynamics simultaneously allow promiscuity and specificity in binding. The multiple conformations of the free EphA4 ligand-binding domain observed in two new EphA4 crystal structures provide a unique insight into the conformational dynamics of EphA4 and its signaling pathways. The heterogeneous ensemble and loop dynamics explain how the EphA4 receptor is able to bind multiple A- and B-ephrin ligands and small molecules via conformational selection, which helps to fine-tune cellular signal response in both receptor and ligand cells.

See research article http://www.biomedcentral.com/2046-1682/5/2

## Commentary

Cell proliferation, differentiation, migration and adhesion are essential processes in development, morphing cells into critical anatomical structures. When cells approach each other, they may have a seemingly simple choice between adhesion and repulsion; however, the precise positioning of cells, as in the case of vascular patterning or in controlling axon growth in the assembly of topographic neural maps, is highly complex.

## The conformational dynamics of cell:cell signaling

Orchestrating the subtle signal transduction regulating these events is performed by two membrane-anchored hub protein families: the Eph receptor tyrosine kinases and their ephrin ligands. To accomplish this complicated task, ten EphA and six EphB receptors evolved to interact with six ephrin-A and three ephrin-B ligands, respectively, effectively maximizing the number of combinations of Eph-ephrin interactions while still maintaining specificity, a principle often encountered in evolution.

The interactions between the Eph receptors and ephrins of the same subclass are promiscuous; however, cross-subclass binding is observed only for two receptors. EphA3 can bind ephrin-B2, and EphA4 interacts with all nine ephrin ligands, each of which has a different function. Previously, nine EphA4 ligand binding domain (LBD) conformations in complex with the ligands were observed in nine crystal structures. This structural heterogeneity of EphA4 can facilitate cross-subclass ephrin signaling[1].  However, unexpectedly, two new crystal structures of EphA4 revealed eight unique conformations in each crystal structure[2].  These snapshots of multiple conformations of the free EphA4 LDB provide a unique insight into the conformational dynamics of EphA4 and the Eph-ephrin signaling pathways.

## Multiple confirmations, multiple ligands

Based on the loop conformations near the binding site, the newly observed EphA4 LBD structures together with those previously known fall into two groups representing open and closed states, indicating the highly dynamic receptor conformations. The protein conformational dynamics were further characterized by molecular dynamics (MD) simulations and nuclear magnetic resonance (NMR) experiments [2]. MD simulations are a powerful tool to explore conformational dynamics (recently reviewed in [3] ). Here the simulations confirm that the loops have much higher intrinsic dynamics than the rest of the structure, and suggest that in the absence of the ligand the open form is less stable than the closed.

Of particular interest, the two forms are dynamically separated by a high barrier and the loops play a key role in the conformational switching between them. Loop dynamics can have two roles: (1) they allow direct interaction with multiple different ligands; and (2) correlated loop fluctuations help in transmitting signals across proteins and their assemblies. The heterogeneous ensemble and loop dynamics explain how EphA4 is able to bind multiple A- and B-ephrin ligands and small molecules via conformational selection.

Three theories have been proposed to explain protein-ligand interactions in signal transduction. The first, the ‘lock and key’ mechanism, considered the protein a rigid molecule that requires an exact conformational match with its ligand to form a functional complex. The ‘lock and key’ mechanism is not applicable to the heterogeneous Eph-ephrin recognition and is unable to explain the modulation of the signal transduction in the Eph-ephrin pathway. The second, the ‘induced fit’ hypothesis, argues that protein complexes often have different conformations from those of their unbound protein constituents because those bound conformations are ‘induced’ by the binding partner. However, what Qin and colleagues[2] observed is that the heterogeneous free EphA4 conformations (including both open and closed loop conformations) already exist before binding to the ephrin ligands.

Over a decade ago, we proposed a third theory, that of the ‘conformational selection and population shift’ [4].  This model recognized that, in reality, biological macromolecules exist in ensembles of conformations for which it suggested distinct functions have evolved; that the ensembles are dynamic; and the populations of conformational species have become optimized and tuned for cellular life. The model further recognized that both binding partners involved in protein-protein interactions are flexible and pre-exist in a range of conformations. During binding, the protein conformers that are most complementary to some pre-existing ligand conformations are/may be preferentially bound. As these conformations bind to their partners, they are removed from the pool of free protein. This disturbs the equilibrium between the different conformations that governs their relative abundance, and other conformations now  undergo a conformational change (a ‘population shift’), so that the equilibrium is restored [4].  Therefore, protein conformational dynamics can pre-encode functional regulation and signal transduction.  The report by Qin and colleagues, which includes a combination of X-ray structures, molecular dynamics simulations, and NMR experiments, provides direct support for the conformational selection mechanism in signal transduction [2].

## Promiscuity and specificity can coexist

Protein conformational dynamics allow promiscuity, but at the same time dynamics can spell specificity. Proteins are often able to bind specifically to more than one partner at the same binding site, a property that has been termed ‘promiscuity’. The partners are often related, and belong to the same family; however, their conformations may vary to some extent. Alternatively, they may belong to different families but share a protein-protein recognition domain, or a motif. Promiscuity is possible because of protein flexibility. In the case of EphA4, the multiple conformations that pre-exist in the free state allow EphA4 to bind nine ephrin ligands.

To understand how conformational dynamics can allow for both promiscuity and specificity, let us consider ubiquitin, for which there are ample data relating to the ‘conformational selection and population shift’ binding mechanism that we have proposed [4].  Similar to EphA4, a large number of conformations has been observed experimentally for ubiquitin, both in solution and in the crystal form, in the unbound state and bound to a range of ligands. In solution, NMR experiments identified an ensemble of 40 conformers. Each of the 40 were mapped to one of the crystal structure conformations in the unbound state or bound to a ligand[5], illustrating that these 40 conformations reflect the inherent population rather than being induced by the ligand. While all conformations were observed simultaneously in solution, this is not the case in the static crystal structure environment, which ‘traps’ a certain favored conformation that has a higher abundance under the crystallization conditions. After mutual conformation selection by ubiquitin and the ligand, minor adjustments of the interactions (induced fit) may take place[6].

**Figure 1:**
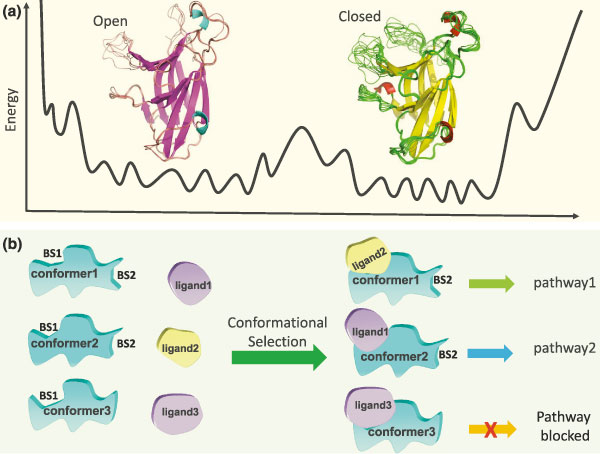
The populations are dynamic and are affected by various factors, including binding. Ligand binding could allosterically affect a second binding site. Because different ligands can allosterically lead to different (second site) conformations and these sites can then select other partners, the effects can propagate downstream through altered signal transduction pathways. **(a)** A high energy barrier may separate the open and closed conformations, as observed for the Eph receptor[2] Sixteen Eph receptor conformations co-exist in two crystals, with eight in each asymmetric unit. Eleven of these can be classified as closed conformations, and five as open. **(b)** Different conformations of the protein can bind multiple ligands via conformational selection. Protein conformational change at one site (BS1) is cooperatively coupled with a change in another site (BS2), in the same or another domain. The conformational selection in BS1 can be coupled with the conformational changes in the BS2. In turn, these conformational changes in BS2 can influence signal transduction pathways through subsequent binding events. The open and closed Eph conformations are taken from Figure 2b,c in [2], with permission from *BMC Biophysics*

The dynamics and the distribution of possible conformations of a protein are encoded in the sequence; these combined with specific residues at the binding site encode binding specificity. It was proposed that the population shift may occur prior to ligand-receptor binding, when the receptor is 1 to 2 nm from ubiquitin[7].  Conformational transitions are typically described by the free energy landscape. As Figure 1 illustrates, the energies of the EphA4 conformational substates are separated by low (within open or closed conformation basins) or high (between open or closed conformation basins) barriers. The ligands match these subtly different conformations. Thus, ligands may have altered binding affinities, allowing specificity across substates. Previously it was shown that the binding affinities of EphA4 with ephrin-A1, ephrin-A2, ephrin-A4, ephrin-A5, and ephrin-B2 are 1.2 µm, 2.3 µm, 36 nm, 360 nm, and 10.8 µm, respectively [1], indicating varied selectivity towards various ligands in solution. *In vivo*, however, the affinities may change.

## Low binding affinity does not mean low functionality

The weak binding affinities of EphA4 across the ephrin-B ligand subclass do not imply functional deficiency. On the contrary, the most prominent examples for EphA4 function are demonstrated through its interactions with this subclass (reviewed in [8]).  The apparent paradox of decoupling of binding affinity from function may also be understood through conformational selection and allosteric regulation. The signal transduction conveyed by the Eph-ephrin binding is bidirectional, relaying the signal into both the receptor (‘forward signaling’) and the ligand (‘reverse signaling’) cells. The signal crosses the membrane to trigger activation of receptor tyrosine kinases as well as other downstream kinases. Long distance signal transduction across the membrane and the cell inevitably involves allosteric regulation through protein conformational dynamics [9].  Protein conformational change at one site is cooperatively coupled with a change in another site, in either the same or another domain. If the energy barrier is low, even a minor perturbation is sufficient to shift the protein conformational ensemble. Long range signaling takes place through multiple allosteric events across the pathway [10].

**Figure 2:**
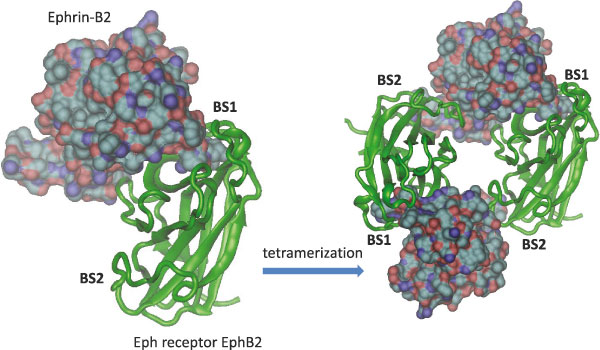
Because both ephrins and Eph receptors have multiple binding sites, however, Eph-ephrin binding may further form hetero-tetramer or higher oligomers if tighter cell-cell adhesion is required [11].  The figure shows the dimeric and tetrameric complexes observed in the crystal structure (PDB: 1KGY).

Mechanistically, signaling involves shifting of the conformations from one state to another. The signal can be in the form of binding of another molecule or, for example, through a post-translational modification event. Such events perturb the protein structure, and the signal propagates via multiple pathways, eventually reaching a second site and changing its conformation or dynamics (Figure 1).  As an example of the coupling of multiple binding sites, Eph-ephrin binding may form hetero-tetramer or higher oligomers [11], which can lead to tighter cell adhesion (Figure 2).  The second binding site on the Eph receptor is also dynamic, but less than the dimer binding site [2].

Signaling is a reflection of the population shift across a protein and across protein-protein interactions, through their binding interfaces. The mutual conformational selection among the protein and ligand conformations across a signaling pathway is not dependent on binding affinity. Higher binding affinity only means that the association of the receptor and ligand is tighter; however, affinity is not necessarily translated into larger conformational change or longer-range allosteric coordination between the ligand binding site and the downstream activation site. Extremely high affinity, normally beyond the biologically occurring functional range, can, however, also trigger an induced-fit binding mechanism. 

## Protein dynamics and conformational selection in signal transduction

Conformational selection and population shift in protein binding and signal transduction are receptor, and ligand, concentration-sensitive. Therefore, it is no wonder that nature widely uses conformational dynamics to allow a graded signal response to stimuli[9].  Eph-ephrin binding can lead to cell repulsion or adhesion. Within this framework, between these two extreme responses, Eph-ephrin-guided cell positioning depends on their ability to assemble into signaling complexes according to the concentration and affinities of the Ephs and ephrins [8].

The conformational states and dynamics observed for EphA4 can help in furthering understanding of the allosteric signal relay in Eph-ephrin signal transduction. These same principles apply more broadly to signaling within and across cells. Examples include the ubiquitination pathway, where E3 ligases mediate ubiquitin transfer from the E2 conjugating enzymes to the substrates. An E2 can bind multiple E3s, and analysis of E2-E3 complexes suggested that loop L1 of E2s is critical. Slightly different conformations of the loop can lead to different specific interactions with E3s, and in this way distinguish between HECT E3s and RING-finger type E3s. Even in the presumably inert cullin scaffolding proteins, it was observed that different loop lengths in the amino-terminal domains confer different dynamical behavior, which allosterically affects the binding site, and thus the choice of partner. Dynamics were also shown to play a prominent role in the protein kinase hub proteins. In protein kinase A (PKA), cAMP acts as a dynamic and allosteric activator, coupling the two lobes of apo PKA, and priming the enzyme for catalysis. NMR and crystallography indicated that a conformational selection rather than an induced-fit mechanism governs substrate recognition [12].

Signaling across long distances is a multistep pathway [13].  Many of the molecules along the pathway have multiple partners, which bind through the same site. Which partner molecule is selected at a given time is critical in deciding the cellular response. Qin and colleagues [2]provide data that clearly illustrate that the binding sites have multiple pre-existing conformations that can select the partner. The principles described for the Eph/ephrin pathway are general, and can be expected to apply to other signaling pathways.

## References

[B1] Bowden TA Aricescu M Nettleship JE Siebold C Rahman-Huq N Owens RJ Stuart DI Jones EY Structural plasticity of eph receptor A4 facilitates cross-class ephrin signaling. Structure 2009 17 1386 1397 10.1016/j.str.2009.07.018 19836338 PMC2832735

[B2] Qin H Lim L Song J Protein dynamics at EphA4 receptor-ligand interfaces as revealed by crystallography, NMR and MD simulations. BMC Biophys 2012 5 2 10.1186/2046-1682-5-2 PMC3274464 22277260

[B3] Durrant JD McCammon JA Molecular dynamics simulations and drug discovery. BMC Biol 2011 9 71 10.1186/1741-7007-9-71 22035460 PMC3203851

[B4] Ma B Kumar S Tsai CJ Nussinov R Folding funnels and binding mechanisms. Protein Eng 1999 12 713 720 10.1093/protein/12.9.713 10506280

[B5] Lange OF Lakomek NA Fares C Schroder GF Walter KF Becker S Meiler J Grubmuller H Griesinger C de Groot BL Recognition dynamics up to microseconds revealed from an RDC-derived ubiquitin ensemble in solution. Science 2008 320 1471 1475 10.1126/science.1157092 18556554

[B6] Wlodarski T Zagrovic B Conformational selection and induced fit mechanism underlie specificity in noncovalent interactions with ubiquitin. Proc Natl Acad Sci U S A 2009 106 19346 19351 10.1073/pnas.0906966106 19887638 PMC2780739

[B7] Long D Brushweiler R In silico elucidation of the recognition dynamics of ubiquitin. PLoS Comput Biol 2011 7 e1002035 10.1371/journal.pcbi.1002035 21533067 PMC3080845

[B8] Lackmann M Boyd AW Eph, a protein family coming of age: more confusion, insight, or complexity? Sci Signal 2008 1 re2 10.1126/stke.115re2 18413883

[B9] Ma B Nussinov R Amplification of signaling via cellular allosteric relay and protein disorder. Proc Natl Acad Sci USA 2009 106 6887 6888 10.1073/pnas.0903024106 19416924 PMC2678435

[B10] del Sol A Tsai CJ Ma B Nussinov R The origin of allosteric functional modulation: multiple pre-existing pathways. Structure 2009 17 1042 1050 10.1016/j.str.2009.06.008 19679084 PMC2749652

[B11] Himanen JP Rajashankar KR Lackmann M Cowan CA Henkemeyer M Nikolov DB Crystal structure of an Eph receptor-ephrin complex. Nature 2001 414 933 938 10.1038/414933a 11780069

[B12] Taylor SS Kornev AP Protein kinases: evolution of dynamic regulatory proteins. Trends Biochem Sci 2011 36 65 77 10.1016/j.tibs.2010.09.006 20971646 PMC3084033

[B13] Nussinov R How do dynamic cellular signals travel long distances? Mol Biosyst 2012 8 22 26 10.1039/c1mb05205e 21766126 PMC7449263

